# Supplementary material for: Efficient knock-in method enabling lineage tracing in zebrafish
Source: Life Sci Alliance. 2023 Mar 6;6(5):e202301944. doi: 10.26508/lsa.202301944 (PMC9990459; doi:10.26508/lsa.202301944)
Supplement: Supplementary file 2 [file LSA-2023-01944_TableS2.docx]

| **Key Resources Table** | | | | |
| --- | --- | --- | --- | --- |
| **Reagent type (species) or resource** | **Designation** | **Source or reference** | **Identifiers** | **Additional information** |
| Strain, strain background (*Danio rerio*) | Tüpfel long fin (TL) | https://zfin.org/ZDB-GENO-990623-2 |  |  |
| Genetic reagent (*Danio rerio*) | *Tg(ptf1a:GFP)^jh1^* | PMID: 16258076 | ZFIN: ZDB-ALT-070531-2 |  |
| Genetic reagent (*Danio rerio*) | *Tg(ela3l:H2BGFP)* | PMID: 28138096 |  |  |
| Genetic reagent (*Danio rerio*) | *Tg(Tp1bglob:H2BmCherry)^S939^* | PMID: 22492351 | ZFIN:  ZDB-ALT-110503-3 |  |
| Genetic reagent (*Danio rerio*) | *Tg(-3.5ubb:loxP-EGFP-loxP-mCherry)^cz1701^* | PMID: 21138979 | ZFIN: ZDB-ALT-110124-1 |  |
| Genetic reagent (*Danio rerio*) | *Tg(ubb:LOXP-CFP-LOXP-zgc:114046-mCherry)^jh63^* | PMID: 25773748 | ZFIN: ZDB-ALT-151007-31 |  |
| Genetic reagent (*Danio rerio*) | *TgKI(krt92-p2a-EGFP-t2a-CreERT2) ^KI126^* | This paper |  | See Methods |
| Genetic reagent (*Danio rerio*) | *TgKI(krt4-p2a-mNeonGreen) ^KI127^* | This paper |  | See Methods |
| Genetic reagent (*Danio rerio*) | *TgKI(krt4-p2a-mNeonGreen-t2a-iCre) ^KI128^* | This paper |  | See Methods |
| Genetic reagent (*Danio rerio*) | *TgKI(krt4-p2a-EGFP-t2a-CreERT2) ^KI129^* | This paper |  | See Methods |
| Genetic reagent (*Danio rerio*) | *TgKI(nkx6.1-p2a-mNeonGreen) ^KI130^* | This paper |  | See Methods |
| Genetic reagent (*Danio rerio*) | *TgKI(nkx6.1-p2a-mNeonGreen-t2a-iCre) ^KI131^* | This paper |  | See Methods |
| Genetic reagent (*Danio rerio*) | *TgKI(nkx6.1-p2a-EGFP-t2a-CreERT2) ^KI132^* | This paper |  | See Methods |
| Genetic reagent (*Danio rerio*) | *TgKI(id2a-p2a-mNeonGreen) ^KI133^* | This paper |  | See Methods |
| Genetic reagent (*Danio rerio*) | *TgKI(id2a-p2a-mNeonGreen-t2a-iCre) ^KI134^* | This paper |  | See Methods |
| Genetic reagent (*Danio rerio*) | *TgKI(id2a-p2a-EGFP-t2a-CreERT2) ^KI135^* | This paper |  | See Methods |
| Antibody | Anti-GFP (chicken polyclonal) | Aves Labs | GFP-1020 | 1:500 dilution |
| Antibody | Anti-mNeonGreen (mouse monoclonal) | Chromotek | 32F6 | 1:50 dilution |
| Antibody | Anti-tdTomato (goat polyclonal) | MyBioSource | MBS448092 | 1:500 dilution |
| Antibody | Anti-insulin (rabbit polyclonal) | Cambridge Research Biochemicals | Customised | 1:100 dilution |
| Antibody | Anti-glucagon (mouse monoclonal) | Sigma-Aldrich | G2654 | 1:100 dilution |
| Antibody | Anti-vasnb (rabbit crude sera) | PMID: 26492970 | Customised (a gift from Paolo Panza, ) | 1:1000 dilution |
| Antibody | Anti-cdh17 (rabbit polyclonal) | PMID: 27401686 | Customised (a gift from Cao Ying) | 1:1000 dilution |
| Recombinant DNA reagent | *krt92*-donor-p2A-EGFP-t2A-CreERT2 (plasmid) | GeneScript | Synthesized | p2A-EGFP-t2A-CreERT2 flanking by 950 base pairs homologous arms in *krt92* 3’ end |
| Recombinant DNA reagent | *Nkx6.1*-donor-p2A-EGFP-t2A-CreERT2 (plasmid) | GeneScript | Synthesized | p2A-EGFP-t2A-CreERT2 flanking by 950 base pairs homologous arms in *nkx6.1* 3’ end |
| Recombinant DNA reagent | *Nkx6.1*-donor-p2A-mNeonGreen-t2A-iCre (plasmid) | GeneScript | Synthesized | p2A-mNeonGreen-t2A-iCre flanking by 950 base pairs homologous arms in *nkx6.1* 3’ end |
| Sequence-based reagent | long HA_common_FWD | This paper | PCR primer | CTCGGTACCCGGGGATC |
| Sequence-based reagent | Long HA_common_REV | This paper | PCR primer | GCAGGTCGACTCTAGAGGATC |
| Sequence-based reagent | *krt4*_common_FWD | This paper | PCR primer | CCAGTGTCACCACCGTCAGCAGTAAACGCTATGGAAGCGGAGCTACTAACTTCAG |
| Sequence-based reagent | *krt4*_ p2a-mNeonGreen _REV | This paper | PCR primer | CTGTGTCGGCTGGGGGTTTGGACGGGCTTCTCCTTACTTGTACAGCTCGTCCATGC |
| Sequence-based reagent | *krt4*_ p2A-mNeonGreen-t2A-iCre _REV | This paper | PCR primer | CTGTGTCGGCTGGGGGTTTGGACGGGCTTCTCCTTAGTCCCCATCCTCGAGCAG |
| Sequence-based reagent | *krt4*_ p2A-EGFP-t2A-CreERT2 _REV | This paper | PCR primer | CTGTGTCGGCTGGGGGTTTGGACGGGCTTCTCCTTAAGCTGTGGCAGGGAAACCC |
| Sequence-based reagent | *nkx6.1*_common_FWD | This paper | PCR primer | ACACAGCTCTTATCATTCATACGTCGGAAAACGAGAGCTCGGGAAGCGGAGCTACTAACTTC |
| Sequence-based reagent | *nkx6.1*_ p2a-mNeonGreen _REV | This paper | PCR primer | GAAGAATCTGACAACCCATTTCCCGTTTCCTTTTTACTTGTACAGCTCGTCCATGC |
| Sequence-based reagent | *nkx6.1*_ p2A-mNeonGreen-t2A-iCre _REV | This paper | PCR primer | GAAGAATCTGACAACCCATTTCCCGTTTCCTTTTCAGTCCCCATCCTCGAGCAG |
| Sequence-based reagent | *Nkx6.1*_ p2A-EGFP-t2A-CreERT2 _REV | This paper | PCR primer | GAAGAATCTGACAACCCATTTCCCGTTTCCTTTTTAAGCTGTGGCAGGGAAACCC |
| Sequence-based reagent | *id2a*_common_FWD | This paper | PCR primer | ATCACAGAGGACAGCAGGACACTTTACCGTGGAAGCGGAGCTACTAACTTCAG |
| Sequence-based reagent | *id2a*_ p2a-mNeonGreen _REV | This paper | PCR primer | ACATTGTATTTTCAAGAAAACTTACCTGATTACTTGTACAGCTCGTCCATGC |
| Sequence-based reagent | *id2a*_ p2A-mNeonGreen-t2A-iCre _REV | This paper | PCR primer | ACATTGTATTTTCAAGAAAACTTACCTGATTAGTCCCCATCCTCGAGCAG |
| Sequence-based reagent | *id2a*_ p2A-EGFP-t2A-CreERT2 _REV | This paper | PCR primer | ACATTGTATTTTCAAGAAAACTTACCTGATTAAGCTGTGGCAGGGAAACCC |
| Sequence-based reagent | EGFP | This paper | Sequencing primer | CATGTGGTCGGGGTAGCG |
| Sequence-based reagent | mNeonGreen | This paper | Sequencing primer | ACTGATGGAAGCCATACCCG |
| Sequence-based reagent | Chemically synthesized Alt-R®-  Modified_tracrRNA | IDT | tracrRNA |  |
| Sequence-based reagent | Chemically synthesized Alt-R®-  modified_*krt92*_crRNA | IDT | crRNA | 5’-AACCTCGCTCGAGATTGGG (AGG)-3’ |
| Sequence-based reagent | Chemically synthesized Alt-R®-  modified_*krt4*_crRNA | IDT | crRNA | 5’-GTCAGCAGTAAACGCTATT (AGG)-3’ |
| Sequence-based reagent | Chemically synthesized Alt-R®-  modified_*nkx6.1*_crRNA | IDT | crRNA | 5’-AGAGCTCGTAAAAAGGAAAC (GGG)-3’ |
| Sequence-based reagent | Chemically synthesized Alt-R®-  modified_*id2a*_crRNA | IDT | crRNA | 5’-AGGACACTTTACCGTTAATC (AGG)-3’ |
| Sequence-based reagent | Chemically synthesized Alt-R®-  modified_*krt92*_crRNA_linearized_1 | IDT | crRNA | 5’-GAGCTCGGTACCCGGGGATC  (AGG)-3' |
| Sequence-based reagent | Chemically synthesized Alt-R®-  modified_*krt92*_crRNA_linearized_2 | IDT | crRNA | 5’- ATCCTCTAGAGTCGACCTGC  (AGG)-3’ |
| Commercial assay or kit | Wizard SV gel and PCR clean-up system | Promega | A9282 |  |
| Chemical compound, drug | LY-411575 | Selleckchem | S2714 |  |
| Chemical compound, drug | X5050 | Sigma-Aldrich | 506026 |  |
| Chemical compound, drug | Acetaminophen | Sigma-Aldrich | A7085 |  |
| Chemical compound, drug | Opti-MEM | ThermoFisher Scientific | 31985062 |  |
| Peptides or recombinant proteins | Alt-R® S.p. HiFi Cas9 Nuclease V3 | IDT | 1081060 |  |
| Peptides or recombinant proteins | Q5 Hot Star high-fidelity 2× master mix | NEB | M0494S |  |
| Software, algorithm | ImageJ | PMID: 22930834 |  |  |
| Software, algorithm | Fiji | PMID: 22743772 |  |  |
| Software, algorithm | LAS X Version 3.5.5.19976 | Leica |  |  |
| Software, algorithm | R | R 4.0.2 |  |  |
| Software, algorithm | RStudio | Version 1.4.1103 |  |  |
| Software, algorithm | ggplot2 | https://cran.r-project.org/package=ggplot2 |  |  |
| Software, algorithm | ggpubr | https://cran.r-project.org/package=ggpubr |  |  |
| Software, algorithm | IBS | IBS1.0.3 |  |  |
| Other | DAPI | ThermoFisher Scientific | D1306 | (1 µg/mL) |
| Other | Nuclease-Free Duplex Buffer | IDT | 11-01-03-01 |  |
